# Talking to pregnant women about stillbirth

**DOI:** 10.1186/1471-2393-15-S1-A12

**Published:** 2015-04-15

**Authors:** Jane Warland, Pauline Glover

**Affiliations:** 1 University of South Australia, Adelaide, South Australia, Australia; 2 Flinders University, Adelaide, South Australia, Australia

## 

It is recognised that consumer awareness of stillbirth is one strategy, in raft of measures, which may reduce stillbirth cases [1]. Raising awareness of the existence of a health issue is often an important first step to take in reducing cases. For example, as a result of the SIDS risk reduction awareness campaigns, the rate of SIDS in high income countries has reduced by as much as 83% [2]. The outstanding success of the SIDS public education campaigns demonstrates that increasing public awareness, alongside an education campaign about protective behaviors, can result in dramatic reduction in prevalence [3]. Therefore, educating women about incidence of stillbirth and encouraging them to be more aware of protecting their unborn baby in order to minimise their risk, is both a potentially feasible and sensible next step in attempting to reduce the occurrence of stillbirth.

Maternal awareness of stillbirth is pre-dedicated on someone making them aware. This responsibility naturally rests with maternity care providers such as midwives and obstetricians. Stillbirth is generally considered a taboo subject in society but also, of concern, by those providing antenatal care [4]. Unfortunately maternity care-providers often avoid discussing the possibility of stillbirth with women in their care. The reluctance to discuss this kind of poor outcome could be to try to avoid “scaring the woman” however, not to do so is missing an opportunity to educate and alert the woman to adopt behaviours to help keep her unborn baby safe [5].

This presentation reported the results of a research project which aimed to educate midwifery care providers about stillbirth incidence, common risk factors as well as how to raise and discuss stillbirth with women during prenatal care. This was done through the delivery of a half-day education package for midwives which provided participants with information about stillbirth. The workshop also provided an opportunity to practice a range of strategies to assist participants to become confident in raising and discussing the topic of stillbirth. The project used a quasi-experimental approach through use of pre and post intervention surveys to determine the effectiveness of the midwife education campaign.

Seventy-two participants completed the pre-workshop questionnaire with 69 participants completing the post workshop questionnaire and 25 completing the 3-month follow-up questionnaire. Responses at the three times points (pre, post, and 3 months) were compared using either Kruskal-Wallis (interval data) Wilcoxon (ordinal data) or Chi–Square (Nominal data) with significance set at p ≤0.05. There was significant improvement in knowledge of the definition of stillbirth, causes and modifiable risk factors as well as knowledge about fetal movements across the participant group. Regarding participant willingness to discuss stillbirth with pregnant women in their care, prior to the workshop 28% of the participants confessed that they never raised or discussed stillbirth with women in their care with a further 64% revealing that they only discussed this with women “sometimes”. Only 4% stated that they “usually” discussed stillbirth with women and no-one indicated that they “always” did. When asked if they planned to change this answer immediately following the workshop 86% replied “yes” with 4% saying no and another 10% unsure. Three months following the workshop there was a statistically significant change (p≤0.001) in attitude to discussing stillbirth with pregnant women with 16% stating that they always did, 12% citing usually and 56 % selecting sometimes with only 4% stating that they still never did.

The project was very effective in raising awareness of the incidence of stillbirth as well as knowledge of risk factors for stillbirth. We anticipate this type of education could ultimately make a difference to stillbirth rates, because if midwives and other maternity care providers raise and discuss stillbirth with women when they are providing antenatal care then this will in turn result in improved maternal awareness of the possibility of stillbirth. This may well lead to women adopting protective behaviors, such as closely monitoring fetal movements and immediately reporting concerns whilst pregnant.
